# Protein-specific Raman imaging of glycosylation on single cells with zone-controllable SERS effect†
†Electronic supplementary information (ESI) available. See DOI: 10.1039/c5sc03560k



**DOI:** 10.1039/c5sc03560k

**Published:** 2015-10-16

**Authors:** Yunlong Chen, Lin Ding, Wanyao Song, Min Yang, Huangxian Ju

**Affiliations:** a State Key Laboratory of Analytical Chemistry for Life Science , School of Chemistry and Chemical Engineering , Nanjing University , Nanjing 210023 , P. R. China . Email: hxju@nju.edu.cn ; Fax: +86 25 89683593 ; Tel: +86 25 89683593; b Department of Pharmaceutical & Biological Chemistry , UCL School of Pharmacy , University College London , London WC1N 1AX , UK

## Abstract

A zone-controllable SERS effect is presented for Raman imaging of protein-specific glycosylation on a cell surface using two types of newly designed nanoprobes. The signal probe, prepared using a Raman signal molecule and dibenzocyclooctyne-amine to functionalize a 10 nm Au nanoparticle, exhibits a negligible SERS effect and can recognize and link the azide-tagged glycan *via* a click reaction. The substrate probe, an aptamer modified 30 or 40 nm Au nanoparticles, can specifically recognize the target protein to create an efficient SERS zone on the target protein. By controlling the size of the substrate probe to match the expression zone of the protein-specific glycan, an efficient SERS signal can be generated. This method has been successfully used for *in situ* imaging of sialic acids on the target protein EpCAM on an MCF-7 cell surface and for the monitoring of the expression variation of protein-specific glycosylation during drug treatment. The concept of zone control can also be used to measure the distance between glycoproteins on a cell surface. This protocol shows promise in uncovering glycosylation-related biological processes.

## Abstract 2

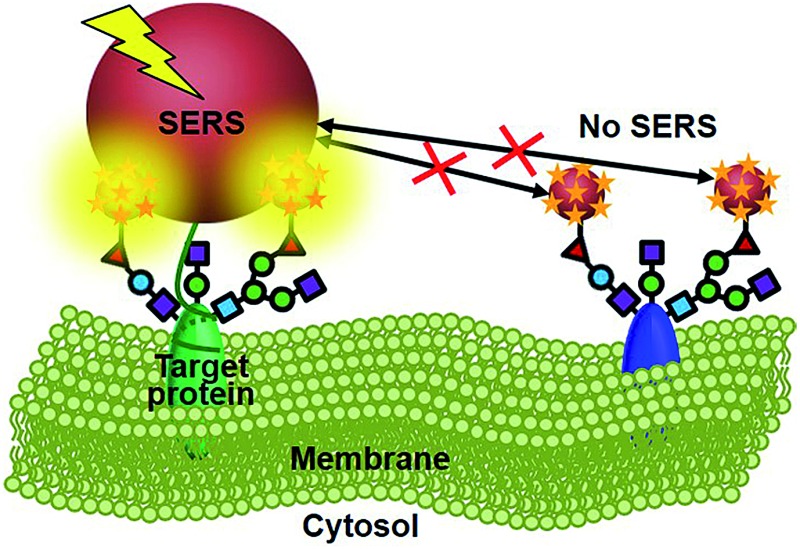
A zone-controllable SERS effect integrates the controlling of nano-substrate size to match the expression zone of protein-specific glycan for Raman imaging.

## Introduction

Glycosylation is one of the most common post-transcriptional modifications of proteins in eukaryotes. Aberrant protein glycosylation profoundly affects cellular adhesion or motility, which further reflects the physiological and pathological states of cells.^1–3^ Thus *in situ* visualization of glycans on specific proteins may provide the correlation of protein glycosylation with disease states and uncover their roles in disease development. Several Förster resonance energy transfer (FRET) methods have been developed for the imaging of protein-specific glycans by labeling the proteins and their corresponding glycans with two FRET-achievable fluorescent molecules.^4–6^ However, one donor to one acceptor FRET mode cannot provide the integral glycan signal on target proteins that are generally modified with more than one glycan molecule. Besides, the short FRET distance between the donor and acceptor^7^ might limit its application in the study of biggish proteins. Thus development of new imaging strategies for monitoring the glycosylation of specific proteins is still in urgent demand.

**Fig. 1:**
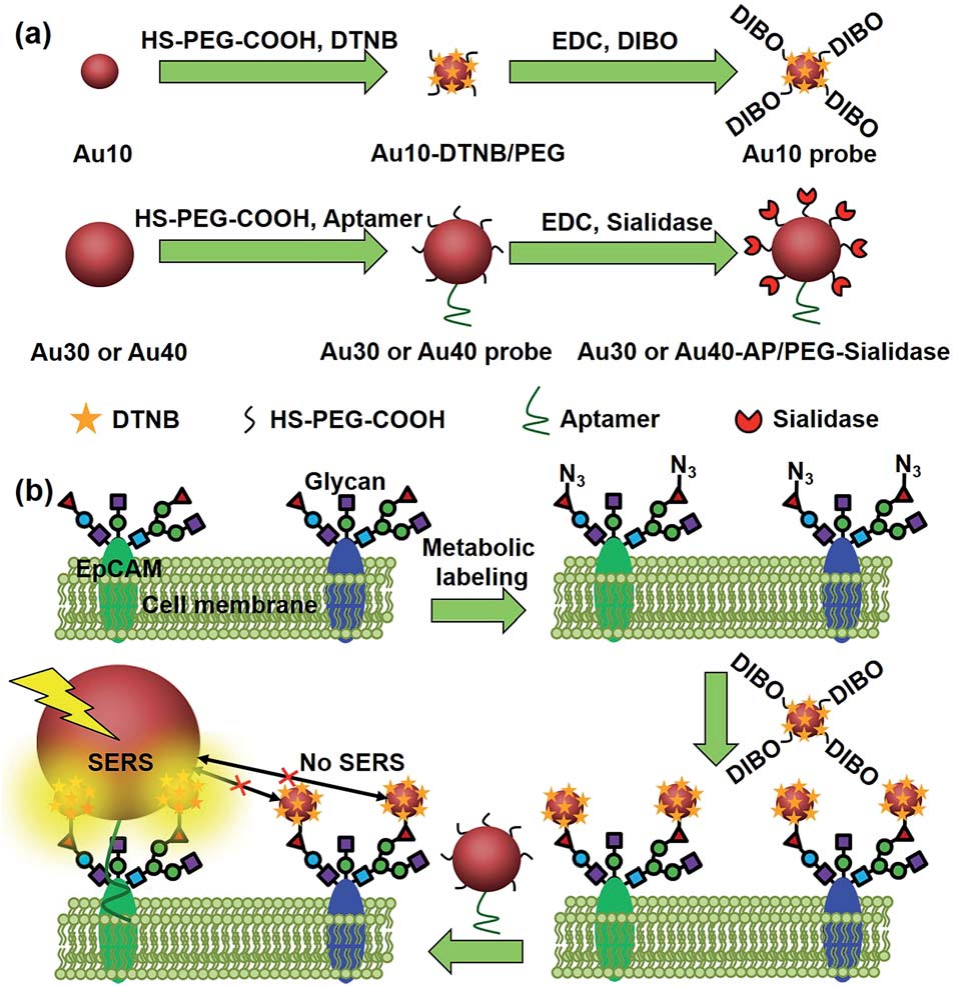
Schematic illustration of (a) the synthesis of two types of Au nanoprobes and (b) the zone-controllable SERS effect for imaging of protein-specific glycans on the cell surface.

Raman imaging based on surface-enhanced Raman scattering (SERS) is a promising non-destructive and non-photobleaching biological imaging technique.^8–10^ It possesses high imaging sensitivity.^11–14^ Different from the FRET, all Raman reporter molecules in the vicinity of the substrate can be enhanced.^15–17^ To provide the exact glycosylation information of the target protein, here we have designed a zone-controllable SERS effect by controlling the size of the substrate to match the expression zone of the protein-specific glycan (Fig. 1), which leads to a strong SERS signal for Raman imaging of protein-specific glycans on the cell surface. Moreover, the concept of zone control can also be used for *in situ* measurement of the distance between glycoproteins on the cell surface.

Generally the optimum size of nano-substrates for SERS is 30–100 nm.^18^ To achieve the zone-controllable SERS effect, Au nanoparticles (AuNPs) with a diameter of 10 nm (Au10), having a negligible SERS effect, were chosen to load the Raman signal molecule, 5,5′-dithiobis (2-nitrobenzoic acid) (DTNB), and 30 nm, the lowest limit for producing the SERS effect,^18^ or 40 nm AuNPs (Au30 or Au40) were used as SERS substrates to select the efficient zone of the SERS effect. The glycan recognition ability of the DTNB-loaded Au10 was achieved using a cyclooctyne terminal (DIBO) with a polyethylene glycol (PEG) linker, which could link with an azide group through copper-free click chemistry.^19,20^ The azide group was formed on the terminal site of the glycan chains by a metabolic glycan labeling technique.^21–23^ The cell surface protein recognition was achieved by modifying the Au30 or Au40 with an aptamer (substrate probe, Au30 or Au40 probe). Here the liberally foldable structure of the aptamer was important for guiding the probe to the site of the target protein.^24–26^ Upon the stepwise recognition of the Au10 probe to target glycan on the target protein and the substrate probe to the protein on the cell surface, two probes approached enough to produce the SERS effect and the Raman signal of DTNB, which could be used for the *in situ* protein-specific Raman imaging of glycosylation on the cell surface. The designed strategy successfully achieved the *in situ* detection of sialic acids on the target protein EpCAM on an MCF-7 cell surface and the monitoring of the expression variation of the protein-specific glycosylation during drug treatment. This work provided a powerful protocol for uncovering glycosylation-related biological processes at a protein-specific level.

## Results and discussion

### Characterization of AuNP probes

**Fig. 2:**
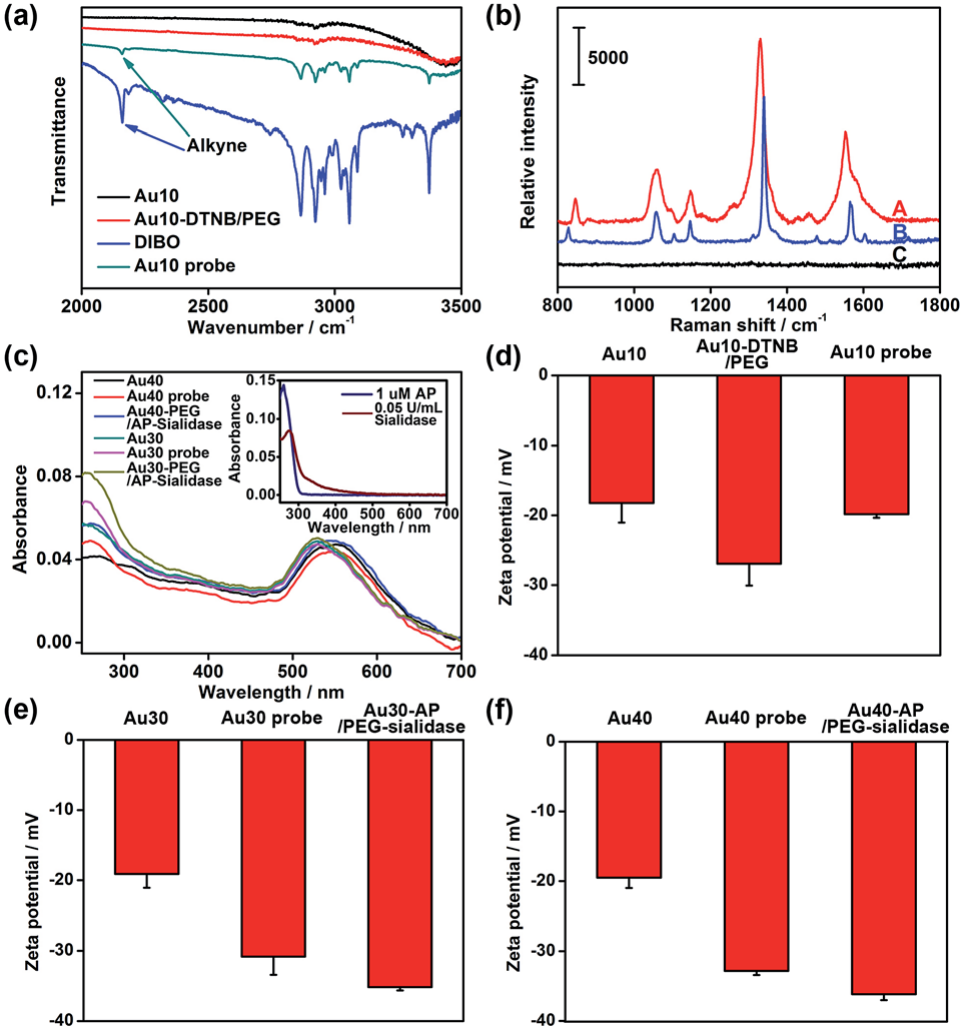
(a) Infrared spectra of Au10, Au10-DTNB/PEG, DIBO and the Au10 probe. (b) Raman spectra of the solid powder of the Au10 probe (A), DTNB (B) and Au10-PEG-DIBO (C). (c) UV spectra of 1 nM Au40, the Au40 probe, Au40-AP/PEG-sialidase, Au30, the Au30 probe and Au30-AP/PEG-sialidase. Inset: UV spectra of 1 μM aptamer and 0.05 U mL^–1^ sialidase. Zeta potentials of (d) Au10, Au10-DTNB/PEG, the Au10 probe, (e) Au30, the Au30 probe, Au30-AP/PEG-sialidase and (f) Au40, the Au40 probe, Au40-AP/PEG-sialidase.

The AuNPs with different sizes were firstly characterized with TEM and dynamic light scattering (Fig. S1†), which showed a narrow size distribution. The Au10 probe showed a characteristic infrared absorption peak of an alkyne group in DIBO around 2160 cm^–1^ (Fig. 2a) and the Raman spectrum was similar to that of DTNB (Fig. 2b), which demonstrated the presence of DIBO and DTNB and indicated the successful synthesis of the Au10 probe. The UV spectra of the substrate probes showed the characteristic absorption peak of DNA at 260 nm (Fig. 2c and inset in Fig. 2c), indicating the binding of the aptamers to Au30 and Au40. The Au30- and Au40-AP/PEG-sialidase showed wider absorbance around 250–290 nm due to the overlap of protein absorbance (Fig. 2c and inset in Fig. 2c), which confirmed the binding of sialidase to these probes. Considering that PEG, the aptamer and sialidase are negatively charged, the zeta potentials with step-by-step change upon each synthesis step of the two types of probes further confirmed their successful modification (Fig. 2d–f). The amounts of aptamer bound on the Au40 and Au30 probes were estimated to be 220 and 150 on each probe by UV measurement of the collected supernatant containing excess aptamer during the probe preparation, respectively (Fig. S2†).

### Verification of the dual-probe system

**Fig. 3:**
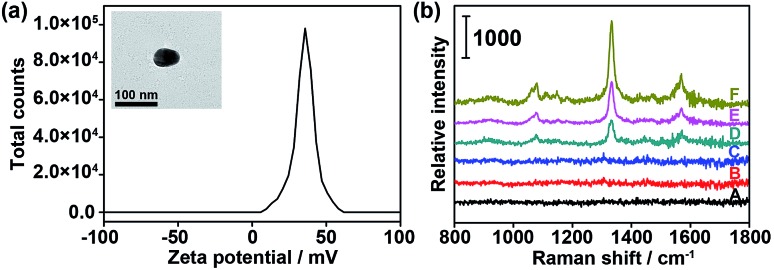
(a) Zeta potential of PDDA-Au40. Inset: TEM image of PDDA-Au40. (b) Raman spectra of (A) 1 nM PDDA-Au40 and (B) 10 nM Au10 probe in PBS, (C) mixture of 10 nM Au10 probe and 1 nM Au40 probe in PBS, (D) 1 nM PDDA-Au40 after incubation with 100 μM DTNB, (E) 10 nM Au10 added in (D), (F) 1 nM PDDA-Au40 after incubation with 10 nM Au10 probe.

To verify the feasibility of the proposed dual-probe nanostructure for generating the SERS effect, positively charged PDDA-Au40 (Fig. 3a) was prepared to simulate the approach of the Au10 probe and the substrate probes to generate the SERS effect. The Raman spectra of both the Au10 probe and the mixture of the Au40 probe and the Au10 probe did not show the characteristic peaks of DTNB (Fig. 3b(B and C)), suggesting the absence of the SERS effect in their free states and the tiny Raman background for Raman imaging. After replacing the Au40 probe with PDDA-Au40, which did not exhibit any Raman response (Fig. 3b(A)), the mixture showed strong characteristic peaks of DTNB due to the electrostatic adsorption of the Au10 probe on PDDA-Au40 (Fig. 3b(F)). This result indicated that the adsorption brought the DTNB and Au40 close to generate SERS and that the designed dual-probe nanostructure can successfully generate SERS when the two-hetero-Au probes are in proximity. The peak intensities were about two times stronger than that of DTNB-adsorbed PDDA-Au40 after further loading with bare Au10 (Fig. 3b(E)), and four times stronger than that of DTNB-adsorbed PDDA-Au40 without the presence of Au10 (Fig. 3b(D)), indicating a greater loading capacity of Raman reporters on the Au10 probe, and a higher SERS efficiency of the dual-AuNP nanostructure formed in the dual recognition process. The dual-AuNP nanostructure could generate stronger plasmonic field enhancements.^27–29^ Thus the dual-probe nanostructure can produce a highly sensitive signal for double recognition triggered high-quality Raman imaging.

### Labeling capability

**Fig. 4:**
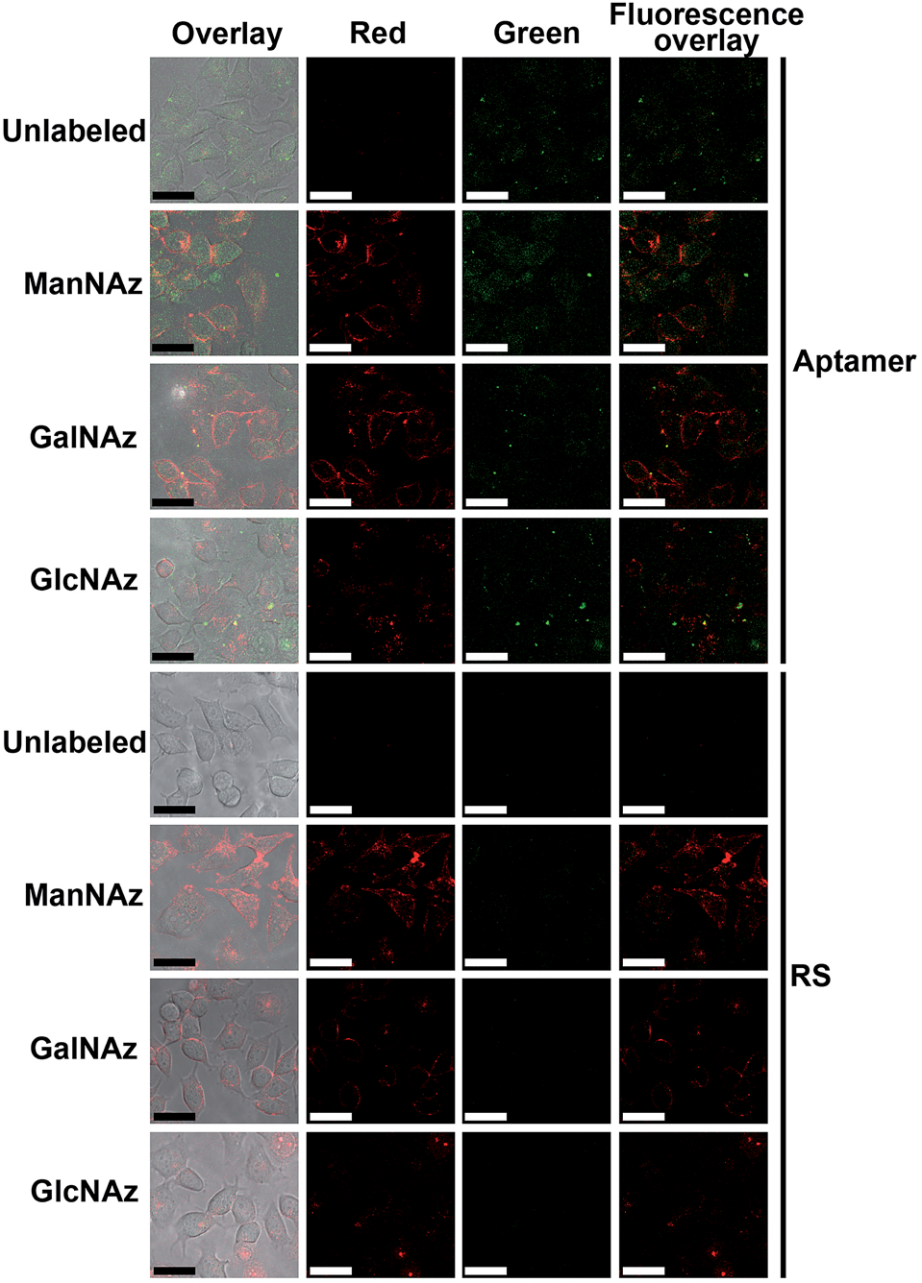
Confocal fluorescence images of unlabeled, and ManNAz, GalNAz and GlcNAz metabolically labeled cells after incubation with 25 μM Alexa Fluor 647 DIBO alkyne for 30 min and then 1 μM FITC-labeled aptamer or random sequence for 30 min. From left to right: overlay, Alexa Fluor 647 fluorescence (red), FITC fluorescence (green) and dual fluorescence overlay images. Scalar bar: 30 μm.

Prior to the Raman imaging of the protein-specific glycans on the cell surface, the labeling capability of the recognition pairs was examined. The epithelial cell adhesion molecule (EpCAM) on human breast cancer MCF-7 cells^30^ was used as the target protein, which is composed of 314 amino acids and contains three N-linked glycosylation sites but no O-linked glycosylation site,^31^ and tetraacetylated N-azidoacetyl-d-mannosamine (ManNAz) was used to metabolically label the cell surface sialic acid (Sia) as the target glycan.^32,33^ Tetraacetylated N-azidoacetylgalactosamine (GalNAz) and tetraacetylated N-azidoacetylglucosamine (GlcNAz), which can metabolically label cell surface O-linked glycans (OLG)^32,33^ and intracellular O-linked N-acetylglucosamine,^33,34^ respectively, were used as negative controls. The existence of EpCAM on MCF-7 cells was firstly confirmed with flow cytometric analyses. The MCF-7 cells exhibited strong binding to both the EpCAM antibody and aptamer, while Ramos cells as the control did not exhibit a fluorescence signal (Fig. S3†). Confocal fluorescence imaging of metabolically-labeled MCF-7 cells was performed with dual-color labeling of EpCAM and azide-labeled glycans using an FITC-conjugated aptamer and an Alexa Fluor 647 DIBO alkyne, respectively (Fig. 4). The images showed overlaid fluorescence signals from the FITC and the Alexa Fluor 647 bound at the cell surface, demonstrating the efficient recognition. However, due to the strong monochrome background the fluorescence intensity was weak, and the overlay of both signals could not provide the linkage information of the glycans with the protein. The specificity of the aptamer-EpCAM recognition was further verified using a FITC-labeled random DNA sequence (RS), which did not exhibit the signal of FITC (Fig. 4). The recognition-mediated adjacent localization of two Au probes on the metabolically labeled cell surface could be observed by TEM images (Fig. S4†). Although the possible crosslinking of several molecules to each probe might happen, it did not affect the monitoring of glycosylation level change of the specific protein.

### Zone-controllable SERS imaging

To obtain high-quality Raman images, the incubation times of three metabolic reagents were optimized to be 48 h using confocal fluorescence imaging with Alexa Fluor 647 DIBO alkyne (Fig. S5†), and the incubation times of two Au probes were optimized to be 30 min by confocal Raman imaging (Fig. S6†). In such a short time the altering of the glycoprotein properties could be neglected.

**Fig. 5:**
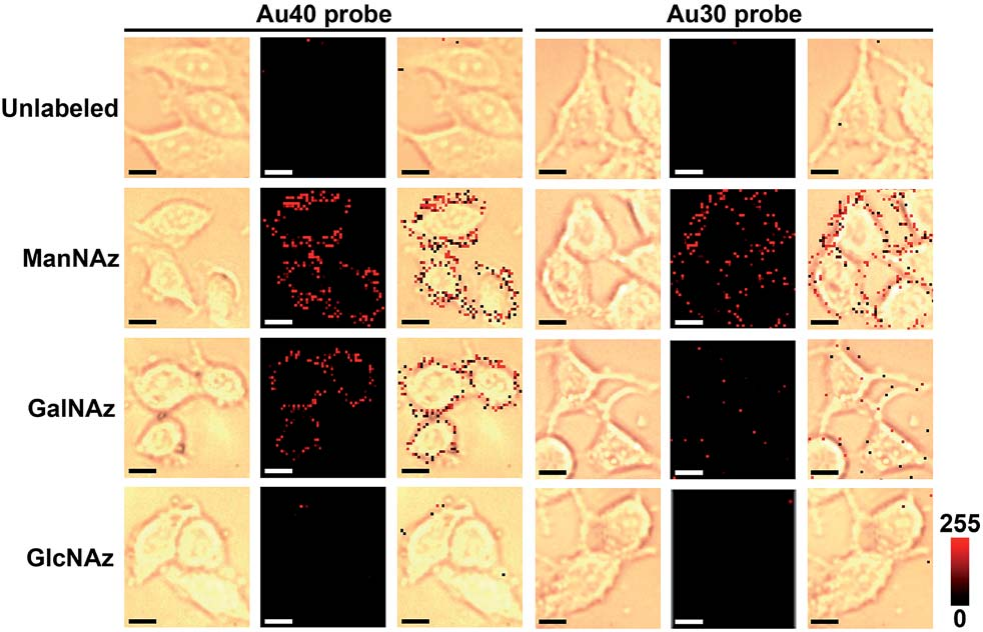
Bright field, Raman and overlay images of unlabeled, and ManNAz, GalNAz and GlcNAz metabolically labeled MCF-7 cells after being treated with different substrate probes. Scalar bar: 10 μm.

Under the optimal incubation conditions, three types of glycans on the cell surface EpCAM were imaged with the zone-controllable SERS strategy using both Au40 and Au30 probes, respectively. The specificity of SERS imaging is mainly decided by the efficient SERS zone of the substrate probe. Chlorpromazine was used as an endocytosis inhibitor during the interaction between the probe and the cells. When the Au40 probe was used, the ManNAz and GalNAz labeled cells showed an obvious Raman signal on the cell surface, which was negligible on the unlabeled cells or in the GlcNAz labeled cells (Fig. 5). In the case of the Au30 probe, only the ManNAz labeled cells showed an obvious Raman signal. The negligible signal on the unlabeled cell surface indicated the binding of the Au10 probe with metabolically labeled cells was specific. Both the negligible signal in GlcNAz labeled cells and the membrane-distributed signal excluded the endocytosis of the substrate probes, which was attributed to the high hydrophilicity of PEG on the probes as well as the short treatment time. Considering the absence of an O-linked glycosylation site on EpCAM,^31^ the signal of GalNAz labeled cells treated with Au10 and Au40 probes could be attributed to the OLG on the neighbouring glycoproteins, thus the absence of a GalNAz signal indicated that the efficient SERS zone of the Au30 probe was appropriate for the zone of glycans expressed on EpCAM, while the Au40 probe was too large. The protein-specific glycan expression zone could be more accurately matched with more kinds of substrate probes to precisely control the size. But a radius of 15 nm for the nano-substrates is the smallest radius for an efficient SERS effect^18^ which limits the detection precision of this method for smallish proteins. FRET based protein-specific imaging^4–6^ is a more appropriate detection technique for a protein with a smallish glycan expression zone.

Since the Au40 probe did interact with the Au10 probe from neighboring non-EpCAM glycoproteins while the Au30 probe did not, the general distance between EpCAM and its neighboring glycoproteins can be estimated to be about 20–25 nm if the minimum requirement for SERS is not considered. Thus the proposed zone-controllable strategy might be potentially used to measure *in situ* the distance between the glycoproteins on the cell surface. Although one report suggested the possible interconversion between GlcNAz and GalNAz by epimerase,^35^ the negligible signal for GlcNAz labelling indicated that the conversion rate was very limited. The signal of Raman imaging kept stable at 24, 48 and 72 hours after the first imaging, which confirmed the stability of the Raman imaging strategy (Fig. S7†).

Compared with the fluorescence images with dual-color labeling (Fig. 4), the Raman images could not only give the glycan information on the specific protein but also exhibited higher intensity and tiny background noise. The EpCAM-negative Ramos cells showed very weak Raman signals for all of the three types of metabolic labeling (Fig. S8†). This further confirmed that only an EpCAM-specific glycan could be imaged using the proposed method. The specificity of aptamer-functionalized probes towards EpCAM was also verified using a RS and PEG co-modified Au40 (Au40-RS/PEG) to replace the Au40 probe, which could not generate an obvious Raman signal (Fig. S8†). This result was consistent with that of the fluorescence imaging (Fig. 4). After the EpCAM knockdown with RNAi experiment,^30^ both of the Raman and fluorescence signals disappeared (Fig. S9†), which further verified the specificity of the protein recognition.

### Monitoring of glycan cleavage and protein-specific glycosylation variation

**Fig. 6:**
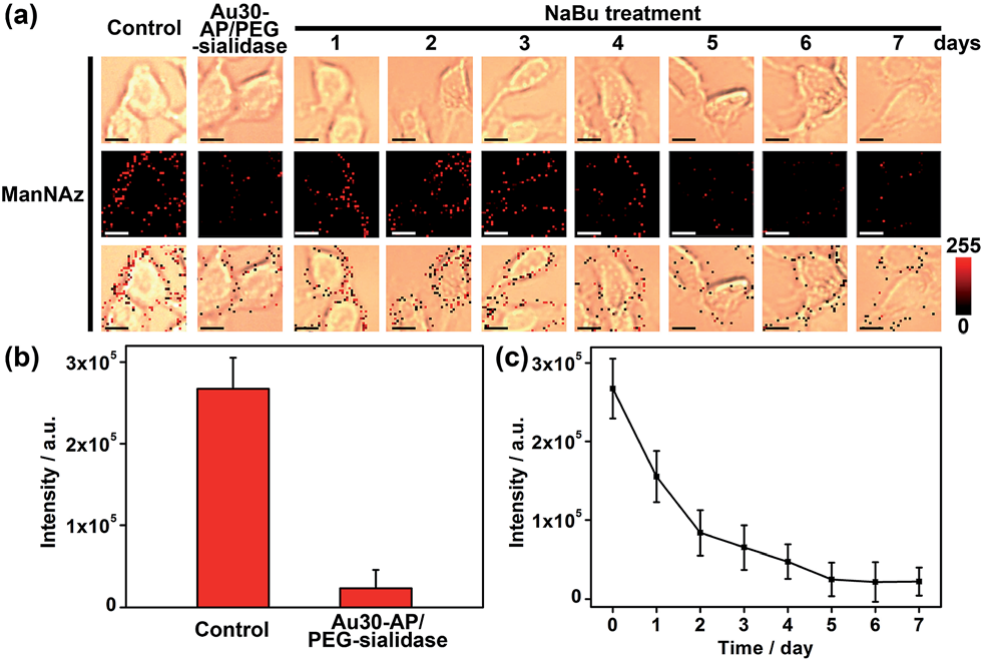
(a) Bright field, Raman and overlay images of ManNAz metabolically labeled MCF-7 cells as control and these cells treated with Au30-AP/PEG-sialidase or NaBu. Scalar bar: 10 μm. (b) Raman intensity obtained from (a). (c) Plot of Raman intensity *vs.* NaBu incubation time.

Since the Au30 probe is more size-appropriate for EpCAM-Sia imaging, the proposed strategy could be used to cleave protein-specific glycans by treating them with cleaving nanoparticles, Au30-AP/PEG-sialidase (Fig. 1). After the specific recognition of the Au10 probe to the cell surface Sia, and the aptamer-mediated binding of Au30-AP/PEG-sialidase to the cell surface EpCAM, the cells were incubated in PBS (pH 7.4) for 30 min, during which the bound sialidase could cleave the Sia under the coverage area of the Au30 probe. The confocal Raman images of these cells showed an obviously decreased signal for Sia on EpCAM (Fig. 6a and b). This result shows that the proposed zone-controllable effect could be used as a powerful glycan cleavage tool at a protein-specific level.

**Fig. 7:**
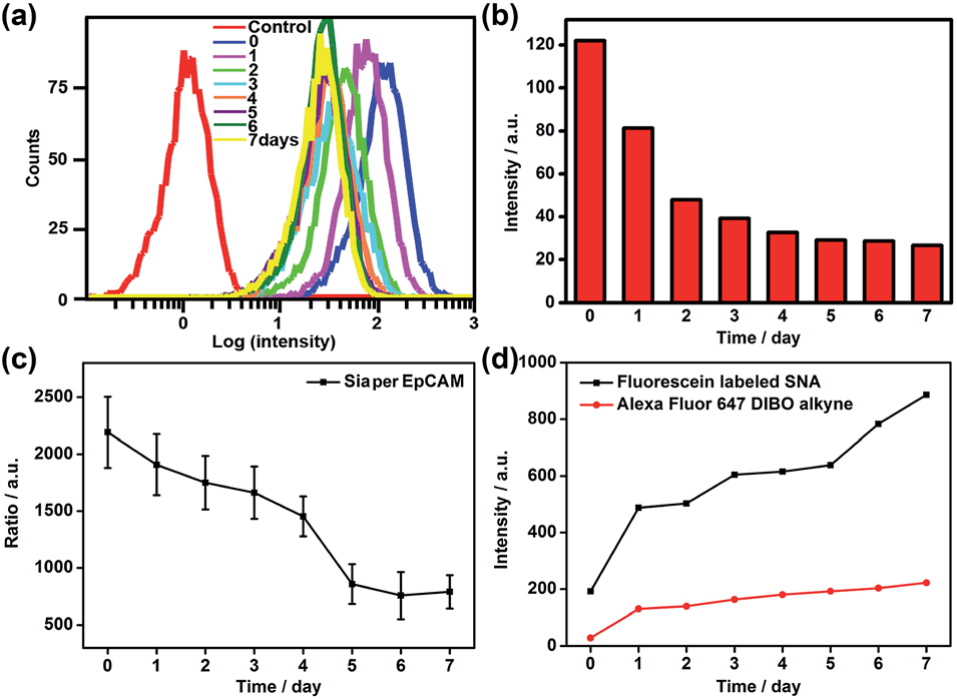
(a) Flow cytometric detection and (b) mean fluorescence intensity of MCF-7 cells treated with 1 mM NaBu for 0–7 days and subsequently incubated with FITC-conjugated EpCAM antibody. (c) Plot of ratio of average Raman intensity of ManNAz metabolically labeled MCF-7 cells after two-probe incubation to corresponding average EpCAM fluorescence intensity *vs.* NaBu treatment time. (d) Plot of mean flow cytometric fluorescence intensity of ManNAz metabolically labeled MCF-7 cells after incubation with fluorescein labeled SNA or Alexa Fluor 647 DIBO alkyne *vs.* NaBu treatment time.

To verify the practicability of the proposed method, MCF-7 cells were firstly treated with 1 mM sodium butyrate (NaBu) for different times, then labeled with ManNAz and recognized by the Au10 and Au30 probes. With the increasing treatment time of 1 to 7 days, the Raman images of these labeled cells showed an obviously decreased signal (Fig. 6a and c). These results indicated the decrease of EpCAM-specific Sia expression on the MCF-7 cell surface, which could be attributed to the down-regulation of EpCAM expression during NaBu treatment^36^ (Fig. 7a and b). By dividing the average Raman intensity corresponding to EpCAM-specific Sia during the NaBu treatment period by the average fluorescence intensity of EpCAM in flow cytometric analysis, the variation trend of Sia expression on each EpCAM protein can be estimated (Fig. 7c). The Sia expression level on each EpCAM showed a decrease with the increasing NaBu treatment time. On the contrary, with regard to the whole cell surface glycan expression detected with the corresponding lectin or Alexa Fluor 647 DIBO alkyne, the NaBu treatment led to an increased Sia expression (Fig. 7d). The increasing expression of Sia on the whole cell surface under NaBu treatment might be due to the up-regulated glycosylation of other glycoproteins.^37,38^ These results indicated that the proposed methods could reflect the glycosylation level change of a specific protein to a certain degree. Thus the strategy based on zone-controllable SERS effect possessed great importance and effective applicability for *in situ* monitoring of protein-specific glycosylation on the cell surface.

## Conclusions

In conclusion, the designed zone-controllable SERS effect has been successfully used for protein-specific Raman imaging of glycosylation by matching the size of the substrate probe with the expression zone of the protein-specific glycan. This effect can be used for the *in situ* monitoring of the cleavage of protein-specific glycans and obtaining the glycosylation variation information of each specific protein. Besides, the conception of zone control could be used for *in situ* measuring of the distance between the glycoproteins on the cell surface. Since Raman imaging can provide detailed spectral information, the proposed method leads to the potential for multi-component research. By combining with other biological labeling technologies, this strategy shows a broad applicability for other proteins which provides a promising protocol for investigation of glycosylation-related biological processes at a protein-specific level.
