# Supplementary material for: Protein-specific Raman imaging of glycosylation on single cells with zone-controllable SERS effect
Source: Chem Sci. 2015 Oct 16;7(1):569–74. doi: 10.1039/c5sc03560k (PMC5519952; doi:10.1039/c5sc03560k)
Supplement: Supplementary file 1 [file SC-007-C5SC03560K-s001.pdf]

Electronic Supplementary Material (ESI) for Chemical Science.
This journal is © The Royal Society of Chemistry 2015

Supplementary Information for

Protein-specific Raman imaging of glycosylation on single cells with zone-controllable SERS effect†

Yunlong Chen,‡[a] Lin Ding,‡[a] Wanyao Song,[a] Min Yang,[b] and Huangxian Ju*[a]

[a]*State Key Laboratory of Analytical Chemistry for Life Science, School of Chemistry and Chemical Engineering, Nanjing University, Nanjing 210023, P.R. China. Fax: +86 25 89683593; Tel: +86 25 89683593; E-mail: hxju@nju.edu.cn*

[b]*Department of Pharmaceutical & Biological Chemistry, UCL School of Pharmacy, University College London, London WC1N 1AX, UK*

‡ Y. L. Chen and L. Ding contributed equally to this work.

Contents

1. Materials and reagents……………………………………………………………S2

2. Apparatus……………………………………………………………………………S2

3. Experimental section………………………………………………………………S3

3.1 Cell culture and metabolic labeling of cell surface glycans………………S3

3.2 Preparation of AuNP probes…………………………………………………S3

3.3 Flow cytometric analysis of cell surface EpCAM and glycans…………S4

3.4 Fluorescence imaging of glycans and EpCAM on cell surface…………S4

3.5 Raman imaging of protein-specific glycans…………………………………S4

3.6 RNA interference……………………………………………………………………S5

4. Supporting figures………………………………………………………………………S5

5. Supporting references……………………………..…………………..…………S10

1. Materials and reagents

Chloroauric acid ($HAuCl_4 \cdot 4H_2O$) was obtained from Shanghai Chemical Reagent Company (Shanghai, China). Trisodium citrate was obtained from Sinopharm Chemical Reagent Co., Ltd. (China). Poly-L-lysine, sialic acid (Sia), 5,5′-dithiobis(2-nitrobenzoic acid) (DTNB), 1-ethyl-3-(3-dimethylaminopropyl) carbodiimide (EDC), poly(diallyldimethylammonium chloride) (PDDA), chlorpromazine, sodium butyrate (NaBu) and dibenzocyclooctyne-amine (DIBO) were purchased from Sigma-Aldrich Inc. (USA). Thiol PEG 1000 carboxyl (PEG) was obtained from Jenkem Technology Ltd. (Beijing, China). Tetraacetylated N-azidoacetyl-D-mannosamine (ManNAz), tetraacetylated N-azidoacetylgalactosamine (GalNAz), tetraacetylated N-azidoacetylglucosamine (GlcNAz), lipofectamine RNAiMAX (Lipo), Alexa Fluor 647 DIBO alkyne and FITC conjugated EpCAM mouse anti-human mAb (clone VU-1D9) were obtained from Life Technologies (USA). Fluorescein labeled Sambucus nigra agglutinin (SNA) was purchased from Vector Laboratories (USA). MCF-7 and Ramos cells were from KeyGen Biotech. Co. Ltd. (Nanjing, China). Tris-EDTA buffer containing 10 mM Tris–HCl and 1 mM EDTA (TE, pH 8.0) was used as DNA stock solution. Phosphate buffer saline (PBS, pH 7.4) contained 136.7 mM NaCl, 2.7 mM KCl, 8.72 mM Na_2HPO_4, and 1.41 mM KH_2PO_4. To reduce the nonspecific binding of aptamer to cells, yeast tRNA (0.1 mg/mL, Sigma) was added to 5 mM $MgCl_2$ contained PBS, which was used as the washing buffer. For temporary inhibition of sialidase, 500 μM Sia was added in the binding buffer (Sia-containing inhibition buffer). All other reagents were of analytical grade. All aqueous solutions were prepared using ultrapure water (≥ 18 MΩ, Milli-Q, Millipore).

The epithelial cell adhesion molecule (EpCAM) aptamer (AP)[S1] was purchased from Sangon Biological Engineering Technology & Co. Ltd. (Shanghai, China) with the sequence: 3'-HS-AAA AAA AAA AAA AAA GTC CGG TTG GGG GGT ACT GTT GCA CCC TGT CTG CGT TGG AGA CAT CAC. The 15 adenines at 3' terminal were the spacer sequence. A FITC-labeled EpCAM aptamer was used for fluorescence analysis. A random DNA sequence (RS) with sulfhydryl or FITC labeled at 3' terminal and the same length of the aptamer was used as the blank control.

The siRNAs were purchased from Shanghai GenePharma Co. Ltd. (Shanghai, China). Human EpCAM siRNA sequences[S2] were 5'-CUACAAGCUGGCCGUAAACdTdT-3' and 5'-GUUUACGG CCAGCUUGUAGdTdT-3'. Control siRNAs were 5'-UUCUCCGAACGUGUCACGUTdTd-3' and 5'-ACGUGACACGUUCGGAGAATdTdT -3'.

2. Apparatus

The transmission electron microscopic (TEM) images were obtained on a JEM-2100 transmission electron microscope (JEOL Ltd., Japan). Dynamic light scattering (DLS) was performed on a 90 Plus/ BI-MAS equipment (Brook haven, USA). The UV-vis absorption spectra were recorded on a Nanodrop-2000C UV-vis spectrophotometer (Thermo, USA). Infrared spectra were recorded on a NICOLET iS10 infrared spectrometer (Thermo, USA). Zeta potential analysis was performed on a

Zetasizer (Nano-Z, Malvern, UK). Flow cytometric analysis was performed on a Coulter FC-500 flow cytometer (Beckman-Coulter). The fluorescence cell imaging was performed on a TCS SP5 laser scanning confocal microscope (Leica, Germany). Raman spectra and images were gained on a Renishaw inVia confocal Raman microscope (Renishaw, UK) using 633 nm excitation. A 50-times telephoto objective was used for observation, spectral measurement and imaging.

3. Experimental section

3.1 Cell culture and metabolic labeling of cell surface glycans

MCF-7 and Ramos cells were cultured in RPMI 1640 medium (GIBCO) supplemented with 10% fetal calf serum (FCS, Sigma), penicillin (100 μg/mL), and streptomycin (100 μg/mL) at 37 ^{o}C in a humidified atmosphere containing 5% CO_2, respectively. The cell numbers were determined using a Petroff-Hausser cell counter (USA).

50 mM metabolic labeling reagents, ManNAz (for labeling cell surface sialic acid), GalNAz (for labeling cell surface O-linked glycans) and GlcNAz (for labeling intracellular O-GlcNAc), were prepared in DMSO as the stock solutions, respectively. 200 μL MCF-7 cells were seeded in a confocal dish at the density of 1×10^5 cells/mL and incubated in medium containing one of the metabolic labeling reagents at 50 μM. The cells were then washed using PBS for 3 times before measurement. Ramos cells were incubated in medium containing the metabolic labeling reagent at 50 μM directly in the cell culture flasks and washed by centrifugation with PBS for 3 times before measurement.

3.2 Preparation of AuNP probes

Three sizes of gold nanoparticles (AuNPs) were prepared with different ratios of $HAuCl_4$ to trisodium citrate[S3] The 10-nm AuNPs (Au10) were synthesized by adding 1.25 mL trisodium citrate (1%) to 50 mL boiling $HAuCl_4$ solution (0.01%), while 30-nm or 40-nm AuNPs (Au30 or Au40) were synthesized by adding 0.67 mL or 0.50 mL trisodium citrate (1%) to 50 mL boiling $HAuCl_4$ solution (0.01%) respectively, which was used to prepare PDDA-coated Au40 (PDDA-Au40) by adding 250 μL PDDA (20%) and 250 μL NaOH (0.5 M) in the mixture. All the mixtures were stirred at 100 ^{o}C until the color became red or purple, and then cooled to room temperature. Prior to use, AuNPs were washed by centrifugation under 12000 rpm for Au10 or 6000 rpm for Au30 and Au40 and resuspended in water. The concentrations of AuNPs were determined using UV-vis absorption spectrometry[S4].

10 μL DTNB (10 mM in ethanol) and 10 μL PEG (1 mM) were added to 1 mL Au10 solution (10 nM) and stirred at room temperature overnight. After washed by centrifugation under 12000 rpm twice and resuspended to 1 mL in PBS buffer, the solution was mixed with 8 μL newly prepared EDC (50 mg/mL) and 10 μL DIBO (50 mM in DMSO), and allowed to react for 4 h. The obtained Au10-DTNB/PEG-DIBO (Au10 probe) was washed by centrifugation under 12000 rpm twice and resuspended to 1 mL in PBS buffer.

100 μL AP (10 μM in TE buffer) and 1 μL PEG (1 mM) were added to 1 mL Au30 or Au40

solution (1 nM) and stirred at room temperature overnight. Afterward, 100 μL NaCl (1 M) was added to the mixtures stepwise within 12 h to stabilize the obtained Au30-AP/PEG (Au30 probe) and Au40-AP/PEG (Au40 probe), which were centrifuged (6000 rpm), washed with PBS twice, and finally resuspended in 1 mL binding buffer, respectively. For specificity demonstration, RS was used to replace AP for obtaining Au40-RS/PEG with the same procedure. To regulate the Sia level on cell surface EpCAM, sialidase was linked to the terminal carboxyl group of PEG through EDC-mediated carbodiimide chemistry by adding 10 μL sialidase (5 U/mL) and 8 μL EDC (50 mg/mL) to 1 mL Au30-AP/PEG or Au40-AP/PEG solution. After reaction for 4 h, the obtained Au30-AP/PEG-sialidase or Au40-AP/PEG-sialidase was washed by centrifugation (6000 rpm) twice and resuspended to 1 mL Sia-containing inhibition buffer.

3.3 Flow cytometric analysis of cell surface EpCAM and glycans

To confirm the existence of EpCAM on MCF-7 cell surface, an EpCAM negative cell line Ramos was used as control. MCF-7 and Ramos cells with concentration of 1×10^6 cells/mL were incubated with 20% FITC-conjugated EpCAM mouse anti-human mAb in PBS or 1 μM FITC-labeled aptamer in binding buffer respectively. After 30-min incubation at 37 ^oC, the cells were washed with PBS or washing buffer twice before flow cytometric test. For investigation of the dynamic change of cell surface EpCAM during NaBu treatment, MCF-7 cells were incubated with 1 mM NaBu for 1 to 7 days and subsequently analyzed with the same procedure. The Sia change on whole cell surface was analyzed by incubating NaBu-treated MCF-7 cells with 0.1 μM fluorescein labeled SNA or 25 μM Alexa Fluor 647 DIBO alkyne in PBS containing 0.1 mM $CaCl_2$ and 0.1 mM $MnCl_2$ for 1 hour at 37 ^oC. The SNA could specifically recognize Sia group on cell surface. Alexa Fluor 647 DIBO alkyne could bind to the metabolically labeled Sia groups on cell surface. The cells were washed with PBS twice before flow cytometric detection.

3.4 Fluorescence imaging of glycans and EpCAM on cell surface

5 mM DMSO solution of Alexa Fluor 647 DIBO alkyne was prepared as a stock solution. To confirm the successful metabolic labeling, 25 μM Alexa Fluor 647 DIBO alkyne in PBS was added to the metabolized cells and incubated for 30 min at 37 ^oC. The cells were then washed using PBS for 3 times. 1 μM FITC-labeled aptamer or random DNA in binding buffer was added to the confocal dishes and incubated for 30 min at 37 ^oC. The cells were then washed with washing buffer for 3 times before confocal fluorescence imaging.

3.5 Raman imaging of protein-specific glycans

The metabolically labeled MCF-7 cells seeded in confocal dishes were first incubated with 200 μL Au10 probe (10 nM) in PBS containing 5 μg/mL chlorpromazine at 37 ^oC. After click reaction between azide and DIBO for 30 min, the cells were washed twice with PBS to remove excess Au10 probe. 200 μL Au30 probe (1 nM) or Au40 probe (1 nM) or Au40-RS/PEG (1 nM) in binding buffer containing 5 μg/mL chlorpromazine was then added to the cells. After 30-min incubation at 37 ^oC, the cells were twice washed with washing buffer and then fixed with 4% paraformaldehyde solution

for 15 min before Raman imaging. As control, the metabolically labeled Ramos cells in the cell culture flasks were treated with the same probe and incubation procedures. After washed by centrifugation, the treated cells were fixed in poly-L-lysine coated confocal dishes with 4% paraformaldehyde solution for 2 h.

The Raman images of cells were obtained by the map image acquisition mode using static scan type at a center wavenumber of 1300 cm^{-1} with exposure time of 1 s, 2 times accumulation and 100% laser power. The imaging step was 1 μm ×1 μm. The strongest characteristic peak of DTNB was at 1330 cm^{-1}, so the Raman images of cells were generated using signal to baseline map review mode from 1300 cm^{-1} to 1360 cm^{-1} by a WiRE 3.4 software, and the color scale of images were chosen as black to red corresponding to the background noise intensity and the maximum signal intensity respectively. The average Raman intensity of cells was obtained from statistics mean value by dividing the total red channel value with the total cell membrane length within the chosen area of the Raman image using Photoshop CS6 software.

The Raman spectra of Au probes were taken by the spectral acquisition mode using static scan type at a center wavenumber of 1300 cm^{-1} with the same parameters as the Raman imaging.

3.6 RNA interference

The MCF-7 cells seeded in confocal dishes were transfected with 10 μM siRNA using Lipo in serum-free medium for 48 hours at 37 ^{o}C according to the manufacturer's protocol. For Raman imaging, metabolic labeling reagents were added together with siRNA. After the cells were twice washed with serum-free medium, they were subject to confocal fluorescence or Raman imaging.

4. Supporting figures

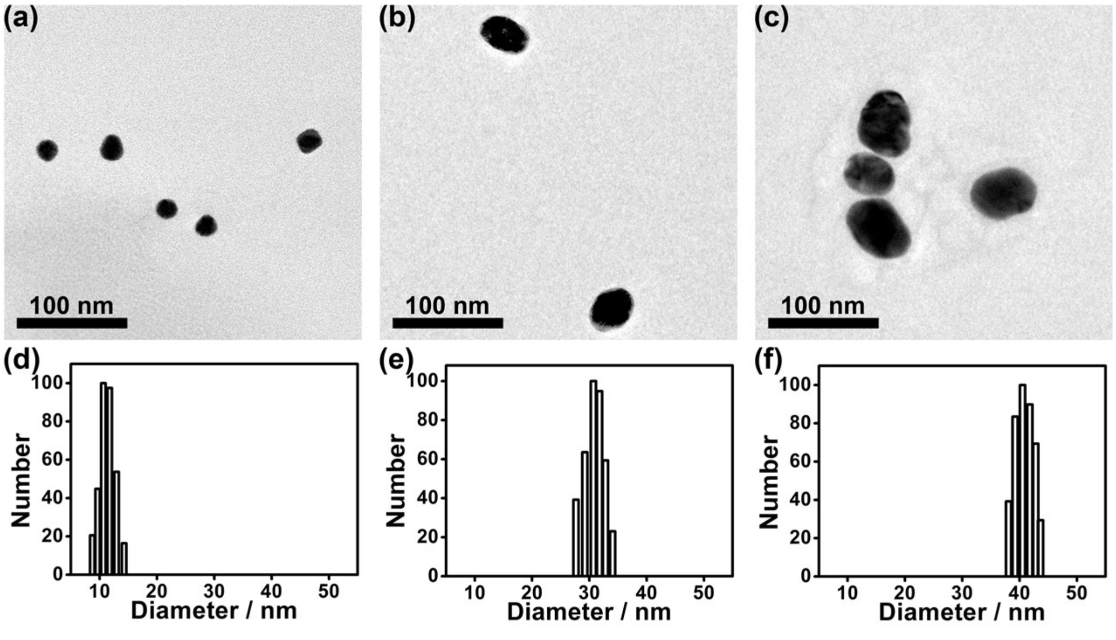

Figure S1 TEM images and DLS of Au10 (a, d), Au30 (b, e) and Au40 (c, f).

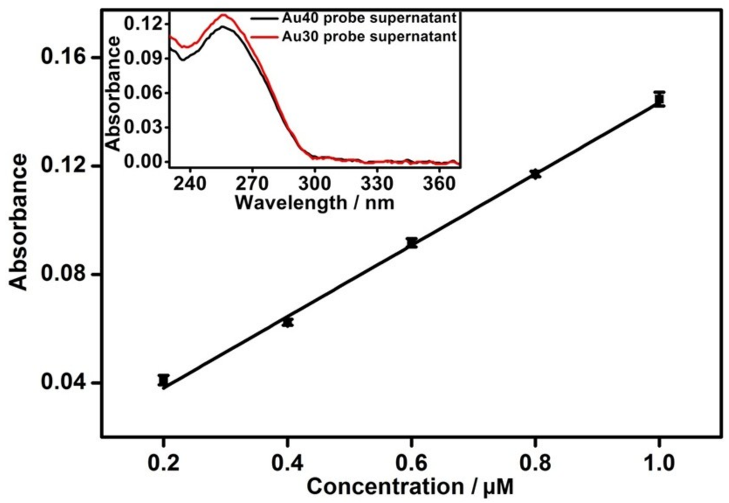

Figure S2 Plot of UV absorbance *vs.* aptamer concentration. Inset: UV absorption spectra of the supernatants containing excess aptamer collected after the preparation of probes.

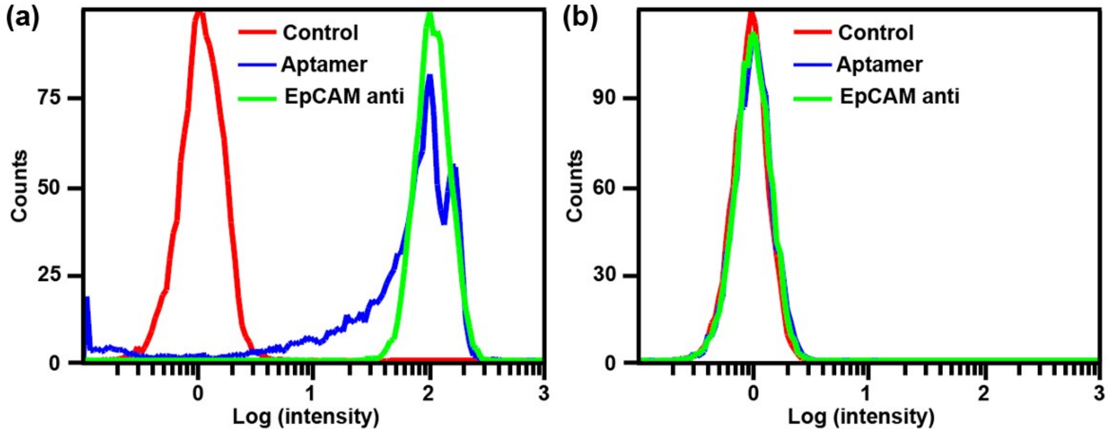

Figure S3 Flow cytometric detection of (a) MCF-7 and (b) Ramos cells (0.5 mL, 1×10^6 cells/mL) after incubation with 1 μM FITC-labeled aptamer or 5-diluted FITC-conjugated EpCAM mouse anti-human mAb (EpCAM anti) using untreated cells as control respectively.

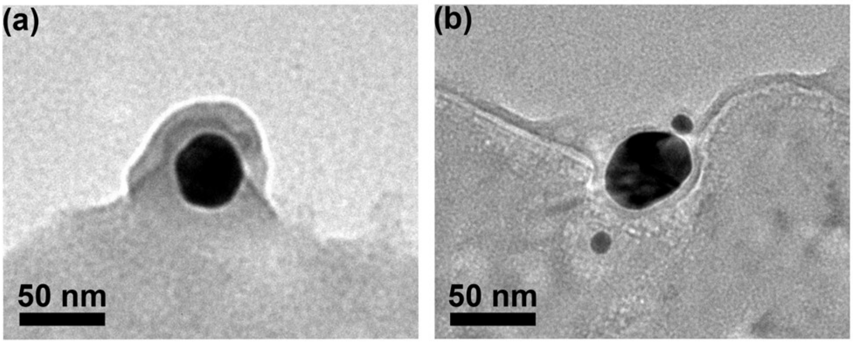

Figure S4 TEM images of (a) MCF-7 and (b) metabolically labeled MCF-7 cell surface after incubation with Au10 probe and subsequent Au40 probe.

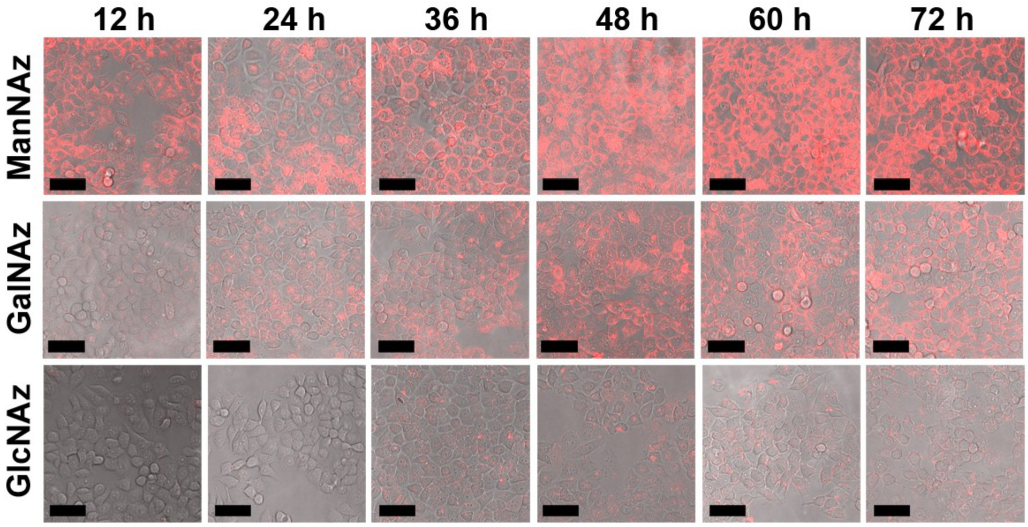

Figure S5 Confocal fluorescence images of MCF-7 cells incubated with 25 μM Alexa Fluor 647 DIBO alkyne for 30 min after different metabolic labeling times of ManNAz, GalNAz and GlcNAz. Scalar bar: 50 μm.

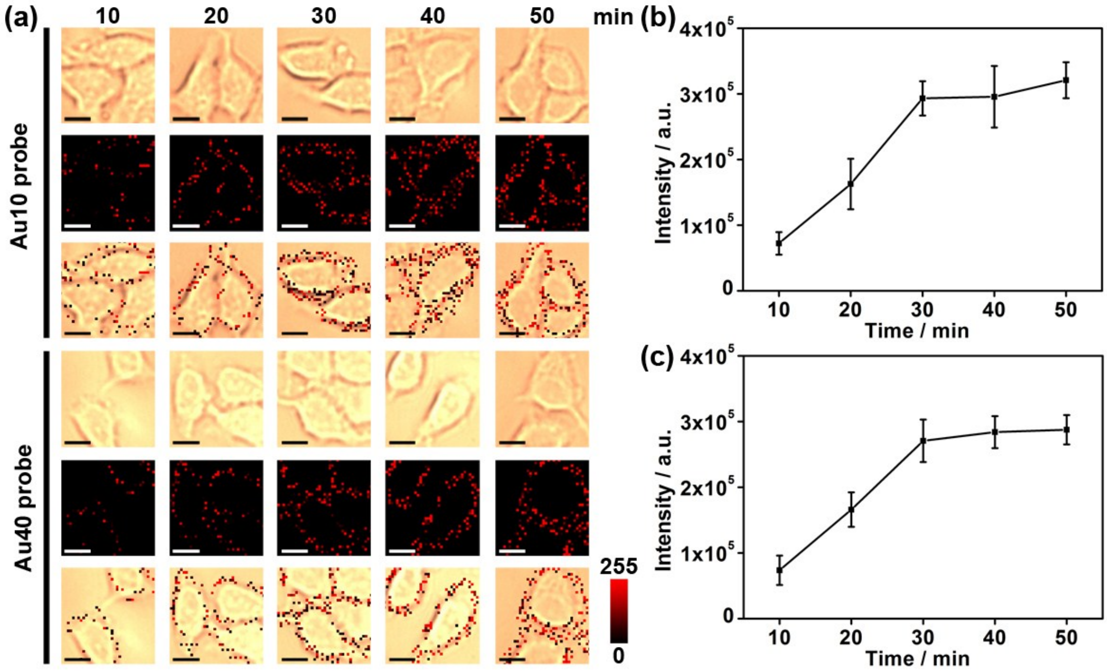

Figure S6 (a) Bright field, confocal Raman and overlay images of ManNAz metabolically labeled MCF-7 cells incubated with Au10 probe and then Au40 probe for marked times, and plots of average Raman intensity of cells *vs.* incubation time with (b) Au10 probe and (c) Au40 probe. When one time changes another time is set at 30 min. Scalar bar: 10 μm.

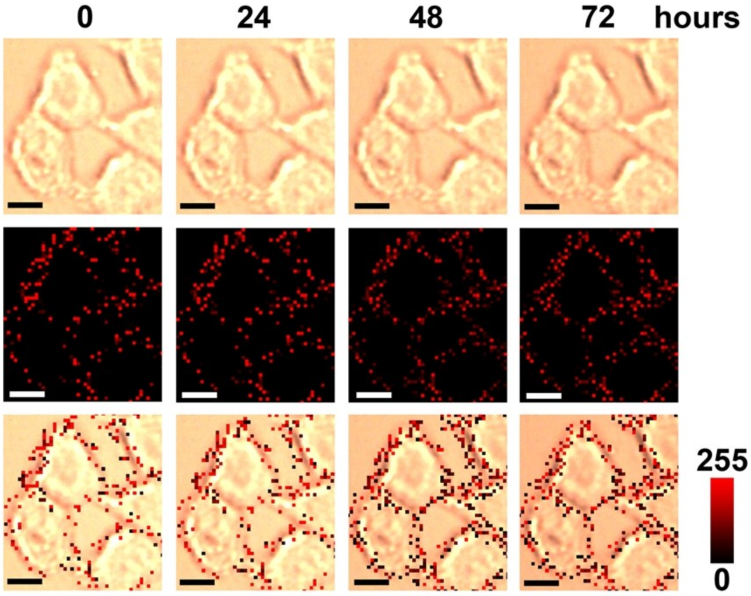

Figure S7 Bright field, Raman and overlay images of ManNAz metabolically labeled MCF-7 cells after 30-min incubation with Au10 probe and then Au30 probe for 0, 24, 48 and 72 hours. Scalar bar: 10 μm.

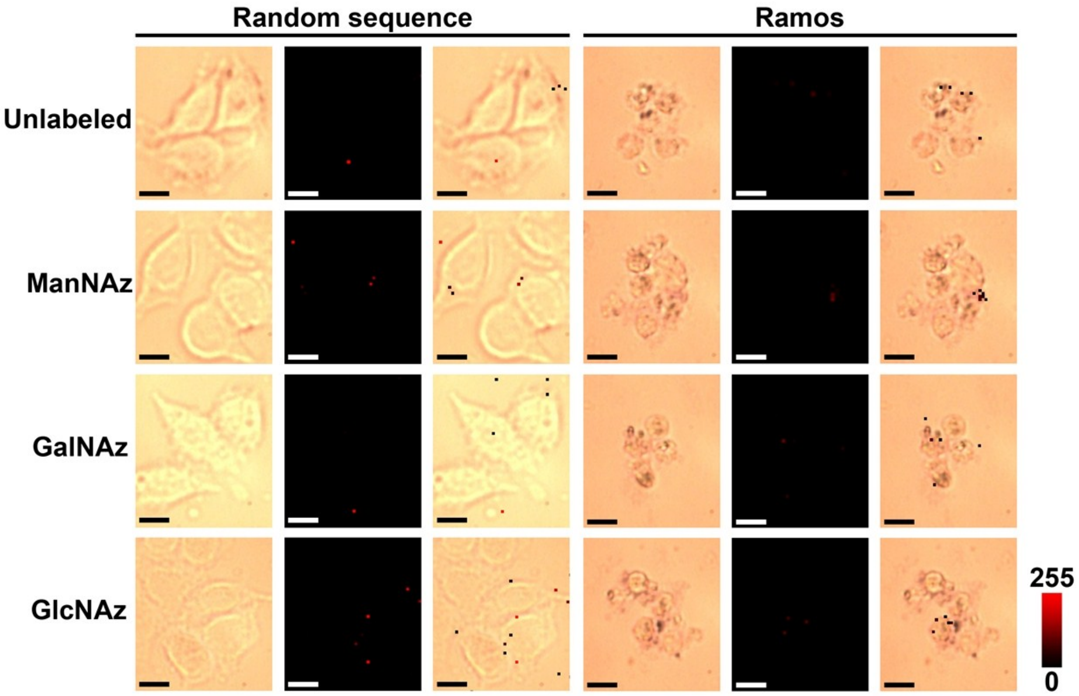

Figure S8 Bright field, Raman and overlay images of unlabeled, ManNAz, GalNAz and GlcNAz metabolically labeled MCF-7 and Ramos cells after incubation with Au40-RS/PEG and Au40 probe, respectively. Scalar bar: 10 μm.

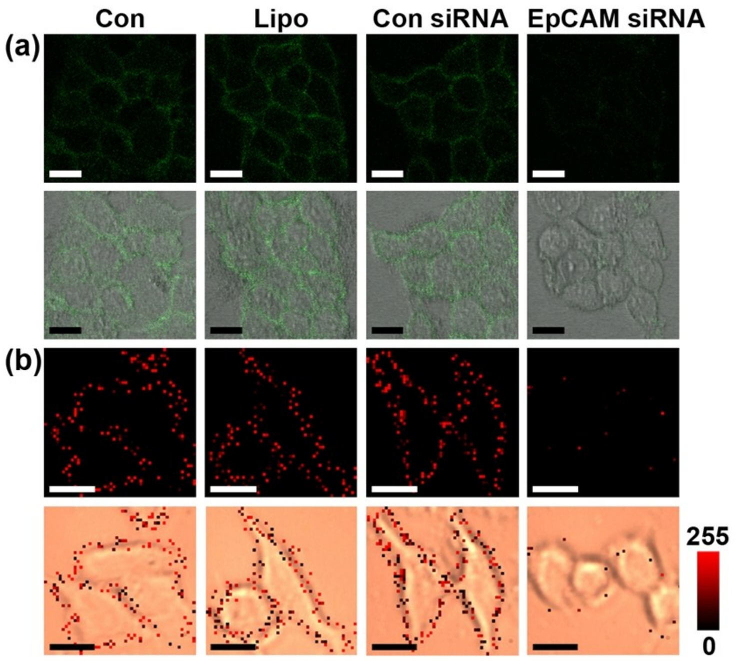

Figure S9 (a) Fluorescence and bright field-overlaid images of control (Con), Lipo, control siRNA (Con siRNA) and EpCAM siRNA transfected MCF-7 cells after ManNAz labeling and 30-min incubation with FITC conjugated EpCAM antibody. (b) Raman and bright field-overlaid images of Con, Lipo, Con siRNA and EpCAM siRNA transfected MCF-7 cells after ManNAz labeling and 30-min incubation with Au10 probe and then Au30 probe. Scalar bar: 15 μm.

5. Supporting references

[S1] Y. L. Song, Z. Zhu, Y. An, W. T. Zhang, H. M. Zhang, D. Liu, C. D. Yu, W. Duan, and C. Y. J. Yang, *Anal. Chem.* 2013, **85**, 4141–4149.

[S2] W. A. Osta, Y. Chen, K. Mikhitarian, M. Mitas, M. Salem, Y. A. Hannun, D. J. Cole, and W. E. Gillanders, *Cancer Res.* 2004, **64**, 5818–5824.

[S3] G. Frens, *Nat. Phys. Sci.* 1973, **241**, 20–22.

[S4] W. Haiss, N. T. K. Thanh, J. Aveyard, and D. G. Fernig, *Anal. Chem.* 2007, **79**, 4215–4221.
